# Nobel prize for the artemisinin and ivermectin discoveries: a great boost towards elimination of the global infectious diseases of poverty

**DOI:** 10.1186/s40249-015-0091-8

**Published:** 2015-12-28

**Authors:** Ernest Tambo, Emad I. M. Khater, Jun-Hu Chen, Robert Bergquist, Xiao-Nong Zhou

**Affiliations:** Department of Biochemistry and Pharmaceutical Sciences, Higher Institute of Health Sciences, Université des Montagnes, Bangangté, Cameroon; Sydney Brenner Institute for Molecular Biosciences, University of the Witwatersrand, Johannesburg, South Africa; Africa Disease Intelligence and Surveillance, Communication and Response Foundation (Africa DISCoR), Yaoundé, Cameroon; Center for Sustainable Malaria Control, Department of Biochemistry, Faculty of Natural and Agricultural Sciences, University of Pretoria, Pretoria, South Africa; Public Health Pests Laboratory, Jeddah Municipality, Jeddah, Saudi Arabia; Department of Entomology, Faculty of Science, Ain Shams University, Cairo, Egypt; National Institute of Parasitic Diseases, Chinese Center for Disease Control and Prevention, Shanghai, 200025 P.R. China; Key Laboratory of Parasite and Vector Biology of the Chinese Ministry of Health, Shanghai, 200025 P.R. China; WHO Collaborating Centre for Tropical Diseases, Shanghai, 200025 P.R. China; Ingerod, Brastad, Sweden

**Keywords:** Nobel Prize, Artemisinin, Avermectin, Ivermectin, Praziquantel, Schistosomiasis, Intestinal helminths, Lymphatic filariasis, River blindness, Malaria, Discovery, Poverty

## Abstract

The Millennium Development Goals (MDGs) made a marked transformation for neglected and vulnerable communities in the developing countries from the start, but infectious diseases of poverty (IDoPs) continue to inflict a disproportionate global public health burden with associated consequences, thereby contributing to the vicious cycle of poverty and inequity. However, the effectiveness and large-scale coverage of artemisinin combination therapy (ACT) have revolutionized malaria treatment just as the control of lymphatic filariasis (LF) and onchocerciasis have benefitted from harnessing the broad-spectrum effect of avermectin-based derivatives. The paradigm shift in therapeutic approach, effected by these two drugs and their impact on community-based interventions of parasitic diseases plaguing the endemic low- and middle-income countries (LIMCs), led to the Nobel Prize in Physiology or Medicine in 2015. However, the story would not be complete without mentioning praziquantel. The huge contribution of this drug in modernizing the control of schistosomiasis and also some intestinal helminth infections had already shifted the focus from control to potential elimination of this disease. Together, these new drugs have provided humankind with powerful new tools for the alleviation of infectious diseases that humans have lived with since time immemorial. These drugs all have broad-spectrum effects, yet they are very safe and can even be packaged together in various combinations. The strong effect on so many of the great infectious scourges in the developing countries has not only had a remarkable influence on many endemic diseases, but also contributed to improving the cost structure of healthcare. Significant benefits include improved quality of preventive and curative medicine, promotion of community-based interventions, universal health coverage and the fostering of global partnerships. The laudable progress and benefits achieved are indispensable in championing, strengthening and moving forward elimination of the IDoPs. However, there is an urgent need for further innovative, contextual and integrated approaches along with the advent of the Sustainable Development Goals (SDGs), replacing the MDGs in ensuring global health security, well-being and economic prosperity for all.

This year's Nobel Prize in Physiology or Medicine, awarded for the discovery of artemisinin and ivermectin, was divided between Youyou Tu "for her discoveries concerning a novel therapy against malaria" and William C. Campbell together with Satoshi Ōmura "for their discoveries concerning a novel therapy against roundworm infections" (Fig. 1). These parasitic infections have endangered human existence disproportionately, impeding productivity and economic growth due to major public health and societal burdens in developing and semi-industrialized countries in Sub-Saharan Africa, Southeast Asia and South America [1, 2]., For example, approximately 25 million people in Africa are still infected by onchocerciasis with more than 300,000 suffering from blindness, which explains the disease's alternative name 'river blindness'. It is estimated that the population at risk of just this one disease in the 31 endemic countries will be 250 million by 2016 [2, 3].

**Fig. 1:**
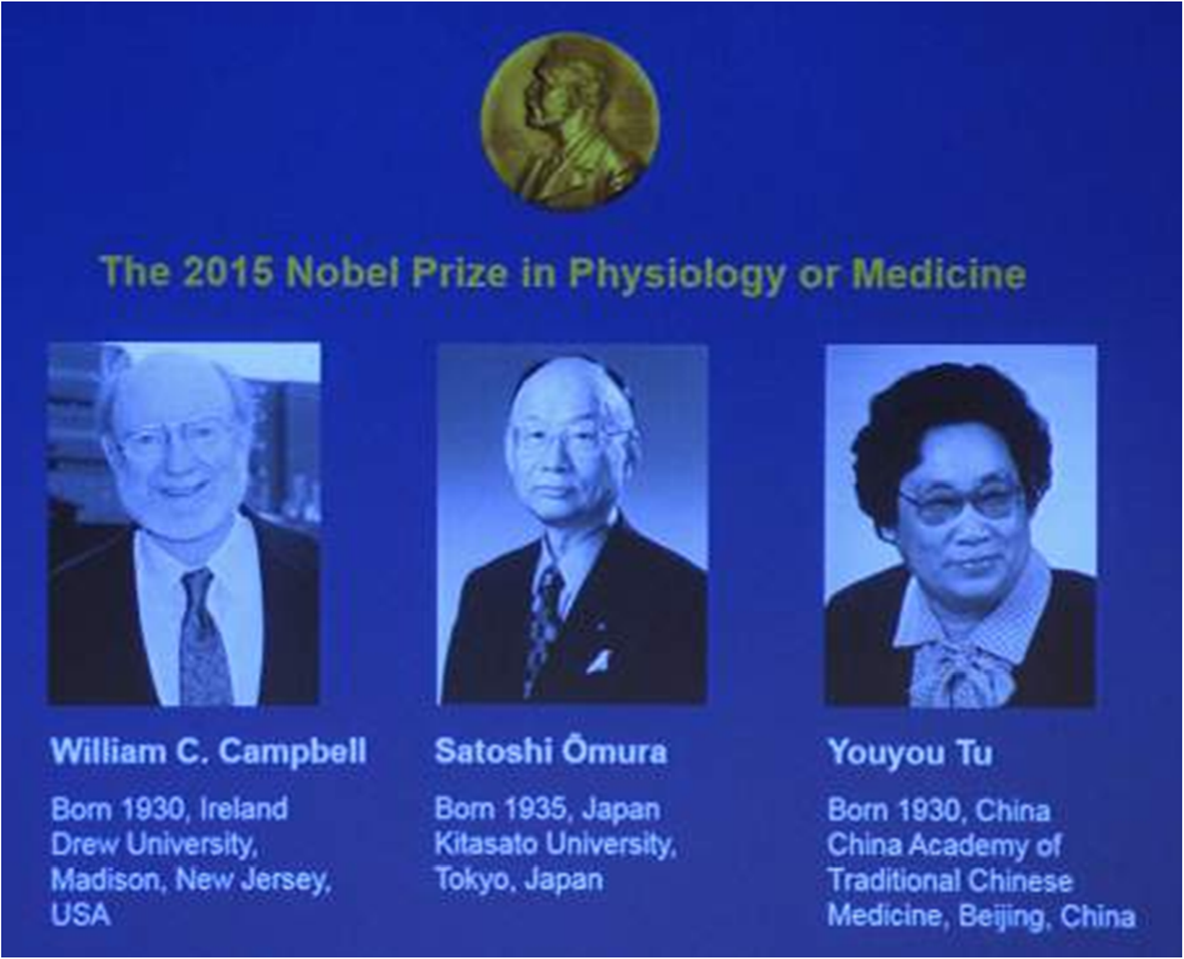
Nobel Prize Laureate Scientists in Physiology or Medicine, 2015

The burden of persisting and threatening infectious diseases in most developing countries is a complex affair, a fact recognized by The United Nations' Millennium Development Goals (MDGs) [4] that represent one of the most successful anti-poverty movements ever undertaken. This 15-year effort to achieve eight goals, set out in the Millennium Declaration of the year 2000, has provided invaluable insights how governments, business and civil society can work together and achieve transformational breakthroughs in many areas, not the least in dealing with long-term, endemic diseases. Ours is a pivotal time for the international development sector with massive implications for global co-operation to control and eliminate endemic diseases and promote transformative social change to end poverty. The new 17 Sustainable Development Goals (SDGs) constitute a continuation and expansion of all aspects of the original eight MDGs and inclusion of neglected tropical disease (NTDs) in the Sustainable Development Goals. With respect to health, the MDG goal number 6 (to combat HIV/AIDS, malaria and other diseases) has been replaced by the SDG goal number 3 (to ensure healthy lives and promote well-being for all at all ages). Goal number one remains the same, i.e. to end poverty everywhere.

The root cause of the infectious diseases of poverty (IDoPs) is the ubiquitous presence of infectious agents. However, social and economic issues play a large role in their transmission and persistence, a fact recognized in both the MDGs and the follow-up SDGs. Still, the transformation of the endemic landscape is almost entirely due to large-scale distribution of the three novel 'wonder drugs' artemisinin, ivermectin and praziquantel, mainly used against malaria, lymphatic filariasis (LF)/river blindness and schistosomiasis, respectively. All three, discovered and developed in the 1970s (though extracts of the plant *Artemisia* - qinghaosu - has a long history in Chinese traditional medicine), are broad-spectrum drugs that can be used to cure many more infections than mentioned above; amazingly, the artemisinins have even effect against immature schistosomes, while ivermectin seems to limit the behaviour of the malaria vector. However, it was not evident that a new era had begun until the new drugs were in general use. Trying to pinpoint this historic shift exactly is of course futile, but it can be said that after the large-scale introduction of modern chemotherapy in the 1980s and 1990s, the first decade of the new millennium marks the time that world-wide implementation of mass drug administration (MDA) occurred [9, 12]. Thanks to market price reductions and broad-scale interventions by international donor programmes, it did not take long until major endemic diseases had become manageable. Indeed, disease control programmes in the endemic countries were not only showing clear progress, but it was also possible to sustain the reduced morbidity and mortality achieved making it realistic to start planning for elimination of many of the endemic IDoPs, a momentous revolution [10, 12].

The discovery of artemisinin and ivermectin have had, and will continue to have, a long-lasting, strong impact on the IDoPs, the former due to its effect on the parasites causing malaria; the latter because its effect on the different nematodes that cause river blindness (*Onchocerca volvulus*), LF (*Brugia* and *Wuchereria*), hookworm infection (*Ancylostoma*, *Necator*) and other soil-transmitted helminthic (STH) infections [1–4]. With the presentation of a road map for the battle against the NTDs in January 2012, the World Health Organization (WHO) inspired the international community to engage into coordinated action involving policy-makers and implementers as well as international donors and the pharmaceutical industry [4]. While it was evident that many of these diseases could realistically be targeted for elimination, it was equally clear that novel drugs, sensitive diagnostics and sustainable approaches for effective surveillance and response together with effective and coordinated, preventive vector control programmes also must be in place [5, 6]. The new drugs, already developed, licensed and used for public health , have decisively demonstrated that malaria, river blindness and LF are preventable diseases whose control would particularly benefit the most vulnerable sectors of the endemic populations [4–8]. Progress has been achieved in all these areas, but greatest impact so far has been with respect to chemotherapy rather than vaccines that will be needed to complement chemotherapy in the longer perspective [7–10].

Youyou Tu's landmark work, documented by increasing access to life-saving, effective artemisinin-based combination therapies (ACTs) against acute and severe malaria, intermittent preventive treatment and curative with respect also to the asexual plasmodium stages, has provided relief and hope for a large proportion of vulnerable populations in the endemic areas [9–11]. Importantly, in the face of increasing resistance of malaria parasites to 4-aminoquinolines (chloroquine) and antifolate drugs (sulphadoxine-pyrimethamine), thanks to the advent of the ACTs that the Global Malaria Action Plan (GMAP) managed to reduce malaria morbidity and mortality by 75 per cent in comparison to the situation in 2005, particularly among the poorest groups across all affected countries aggregately [12, 13]. Moreover, the new chemotherapy approach has made it possible to scale up national malaria control and elimination programmes enhancing interventions and universal health coverage to achieve WHO's Roll Back Malaria (RBM) initiatives and those of the MDGs [13, 14]. For example, by the end of 2012, the United Nations Children's Fund (UNICEF) had procured about 25 million ACTs treatments for 28 countries; however the proportion of children in sub-Saharan Africa with access to ACT is still variable and in many cases despairingly low (range <7 % to >90 % with the latter level only reached in a few countries) [10, 15, 16]. ACT is the drug of choice against acute and severe infection by *Plasmodium falciparum* and *P. vivax*, the most deadly malaria species, which are now resistant to chloroquine and antifolate drugs [10, 11, 16] in most endemic areas. Hence the call for innovative and integrated community-based packages [16, 17] in strengthening implementation and management has executed by the Malaria Eradication Research Agenda (malERA) and the Malaria Eradication Scientific Alliance (MESA) in the most highly endemic developing and semi-industrialized countries [18].

Both acute and severe malaria continues to exert a deep-rooted impact in 109 countries and territories around the world and still constitute a leading risk factor for infant mortality and sub-optimal growth and development in spite of the global malaria elimination campaign programmes (GMECPs) that were in effect in the 1950s to the 1970s [10, 19]. By 2000, there was an estimated 350–500 million cases of malaria and more than one million deaths, most of them in children under 5 years, pregnant women and non-immune travelers in Africa and Asia-Pacific [19]. The saying “One child dying of malaria every second” remains true to this day, and the disease still has a serious economic impact in Africa, retarding economic growth and development and perpetuating the vicious cycle of poverty [15, 18–20].

In 1971, at the Pharmaceutical Institute of the Academy of Traditional Chinese Medicine, Youyou Tu showed that *Artemisia* plant extracts could kill *P. berghei*, a rodent malaria parasite laboratory model. The following year, she succeeded in isolating the active ingredient (qinghaosu), now part of ACT, the most important class of anti-malaria medications [9, 20, 21]. Artermisinin derivatives are postulated to act by inhibiting the major metabolic processes of the malaria parasite, such as glycolysis, nucleic acid and protein synthesis, thus exercising a broad- based activity extending to impeding the development of gametocytes that promises future development of transmission-blocking, sexual-stage drugs and vaccine discovery [5, 6, 11, 15, 22, 23]. Still, years after the original discovery of the drug, the complete mechanism of artemisinin is not fully elucidated, though recent evidence suggest that is based on the activity of 13 proteins, some of which shown to be involved in the emergence of resistance in Southeast Asia, e.g. the Thai-Cambodia borders areas [24]. Impressive progress has been achieved in product development, manufacturing, procurement as well as financial accessibility to treatment (the latter regarding the ACTs in particular) even if prompt, affordable and widespread coverage is still not achieved in many public health facilities in remote, hard-to-reach endemic settings [12, 24, 25]. From 2004 to 2006, the annual global procurement of ACT increased from 4 million to ~100 million doses (~125 million doses in 2007), around 70 % of which were used in Africa, resulting in a significant reduction of morbidity and mortality in children and trimmed-down numbers of acute and severe cases of malaria overall [12, 14, 25, 26].

The latest decades have witnessed substantial progress in raising awareness and increasing the production, adoption and distribution of existing effective interventions besides the use of ACTs, e.g. indoor residual spraying (IRS) and large-scale distribution of long-lasting insecticidal nets (LLINs) as prescribed by the RBM's universal coverage partnership [12, 27, 28]. The latest available worldwide report of malaria cases concerns the year 2013, in which WHO reports that about 3.2 billion people are at risk of this infection with more than 132 million confirmed cases; however, the real level of incidence must be higher since the number of suspected cases has surpassed 367 million [29]. Africa still has the heaviest burden with children less than 5 years old making up 90 % of the malaria-related deaths, which accounts for 78 % of the total mortality [29, 30]. In addition to the high local healthcare burden in Africa, malaria illness and mortality affect crop production and decrease tourism, which is estimated to cost approximately USD12 billion each year due to increased school and work absenteeism, lost productivity and constraints to foreign investment [10, 30]. Hence, although the impact of malaria in the endemic countries has abated to some extent in some areas, transmission continues at a high level assuring that the global ACTs demand will remain robust over the coming years as treatment-adherence and compliance remain vital in the struggle to substantially reduce malaria morbidity and mortality supporting the hope of eventually reaching sustained control, elimination and subsequent eradication of the disease [29, 31].

William C. Campbell and Satoshi Ōmura discoveries proved to be a breakthrough in the tenacious fight against infections caused by roundworm parasites, mainly onchocerciasis and LF, dreaded health scourges of the most vulnerable groups of affected populations. The discovery, industrial development and implementation of the active avermectin derivative ivermectin (best known under the brand name Mectizan) led to a significant reduction of onchocerciasis in the endemic areas in central Africa and Latin America as well as of LF and scabies that are also endemic in India and Southeast Asia, thus improving the situation in vulnerable communities in low- and middle-income countries (LIMCs) [1, 3]. In an unusual move, the manufacturer of ivermectin, Merck & Co., Inc. declared it would donate ivermectin free of charge for as long as it would be needed through its Mectizan Donation Program, which works with governments (Ministries of Health) and various partners with national onchocerciasis control programmes to scale up distribution and coverage of the drug locally [9, 32]. Its impact on mosquitoe control in malaria is debatable and the drug is not donated for this purpose.

Onchocerciasis, transmitted by the filarial worm *O. volvulus*, is transmitted to humans through bites of infected female blackflies (*Simulium* spp.). Adult worms can live up to 18 years in infected human hosts and release up to 1,000 microfilariae daily causing a variety of ailments including skin lesions due to chronic dermatitis, , rashes, intense itching, depigmentation as well as visual impairment due to eye inflammation causing corneal scars eventually leading to irreversible blindness [3, 32]. The disease is endemic in 35 countries, including 28 African countries, Yemen in the Middle East and six Latin American countries resulting in an estimated 17.7 million people infected, approximately 500,000 with visual impairment, 270,000 of whom are blind; about 99 % of all cases, however, are found in Africa according to WHO [2, 32, 33]. The Onchocerciasis Control Programme (OCP) operated in West Africa from 1974 to 2002 [2–33] and its work is being continued by the larger African Programme for Onchocerciasis Control (APOC) that coordinates annual community-wide treatment regimens with ivermectin in 16 countries. An estimated 8.2 million disability-adjusted life years (DALYs) [4, 13] at a nominal cost of about USD257 million was averted between 1995 and 2010 and APOC estimates to have warded off another 9.2 million DALYs between 2011 and 2015 at a nominal cost of USD 221 million [2, 4]. Interventions in countries in West Africa employed large-scale, community-directed treatment with ivermectin (CDTI) and ONCHOSIM, a computer-based software developed to model the transmission and control of onchocerciasis allowing the continuous annual treatment of more than 30 million people [34]. As of 2012, over 200 million people have received ivermectin; 118 million a combination of ivermectin and albendazole and more than 100 million people have been treated in 26 countries in 2013 alone [35].

The Onchocerciasis Elimination Program of the Americas (OEPA) was launched in 1992 under the Pan American Health Organization (PAHO) with the goal of interrupting onchocerciasis transmission in six endemic Latin American countries by 2015 [36]. The WHO's regional NTDs elimination agenda includes fostering coalition and partnerships in resource mobilization aimed at increasing free availability and accessibility of ivermectin to needy populations. Since 2007, WHO also engaged in activities ensuring periodic sustainability of treatment activities and feasibility studies of foci and ongoing regional eradication [36, 37]. Columbia became the first country achieving elimination of onchocerciasis; Ecuador and Mexico have also been verified as free from transmission (verified by WHO in 2013). Likewise, Brazil and the Bolivarian Republic of Venezuela have embarked on reciprocal, cross-border interventions finding ivermectin very effective in controlling the disease [36–38]. Ivermectin is provided as MDA once or twice annually to millions of the most vulnerable children and adults populations in most LMICs [3, 4, 39, 40]. Interruption of transmission of *O. volvulus* and reduction of the burden of visual impairment and blindness have been achieved [41, 42]. However, repeated ivermectin treatment showed reduced susceptibility in the India and South East Asia LF control to elimination programmes. This has led to the call for novel alternative approaches in accelerating and sustaining the transmission-free status once achieved to avoid the risk for re-introduction or resistance of the disease in the process of elimination and eventual eradication in Africa and others areas (e.g.: Yemen) with disease co-endemicity [33, 35, 43].

Though not recognized for this year's Nobel Prize, the story would not be complete without mentioning praziquantel. Praziquantel, a drug that has modernized the control of schistosomiasis and many other helminth infections in the same way the ACTs and ivermectin worked for malaria and LF/onchocerciasis, respectively [44, 45]. Praziquantel was developed by the German pharmaceutical companies Merck KGaA, Darmstadt and Bayer AG, Leverkusen in the early 1970s [46–48] and the drug currently figures on WHO's list of essential medicines [49]. The drug’s good safety profile and broad therapeutic efficacy extend its therapeutic efficacy from the five *Schistosoma* spp. species capable of infecting humans [47, 50–52] to cestodes (tapeworms) such as *Echinococcus* spp. [49], whose larval stages infects various organs, *Taenia* spp. that can infect the brain and muscles with its eggs and larvae (cystocercosis) and food-borne trematodes (FBTs), such as *Paragonimus* spp., *Opistorchis* spp. and *Clonorchis* spp. [50].

FBT transmission is linked to traditional customs, e.g. consumption of dishes containing raw fish, crustaceans and plants in countries where these diseases are sustained by entrenched cultural practices, which are difficult to change. FBTs affect over 56 million people infected in over 70 countries [51]; this figure, however, includes *Fasciola* spp., a parasitic worm preferably treated with triclabendazole (sold under trade names Egaten and Fasinex) [52].

Schistosomiasis is acquired through contact with water infested with cercariae, the free-swimming larval forms emanating from the intermediate snail host when infected. The microscopic adult worms live in the veins of abdominal organs, where large amounts of eggs are produced for excretion via faces or urine aimed at hatching and infecting fresh-water snails thus closing the parasite's lifecycle. Large numbers of the eggs are, however, trapped in the tissues and where immune reactions cause damage that varies from subtle to serious. Millions of people suffer from severe schistosomiasis [2, 44, 53], a type of injury that can be suppressed by regular treatment preventing reinfection giving rise to morbidity. The current WHO chemotherapy-based strategy controls the morbidity in poor and marginalized communities in conjunction with interventions against cestode and nematode infections with albendazole and ivermectin. Severe morbidity due to schistosomiasis can be prevented by regular treatment of risk groups targeted based on community diagnosis at sentinel sites. Like malaria, LF and river blindness, schistosomiasis is prevalent in tropical and sub-tropical areas, in poor communities without potable water and adequate sanitation. Like the other infections discussed here, schistosomiasis is one of IDoPs. It affects about 240 million people worldwide, and more than 700 million people live in endemic areas [53, 54]. In contrast to FBT infections, cysticercosis and echinococcosis, there has been strong progress on the schistosomiasis agenda, mainly thanks to long-term, national control programmes that in some countries, notably Brazil, China and Egypt, have been highly successful in driving prevalence and morbidity down. In 2013, more than 39 million people were treated for this disease with praziquantel. However, this represents only about 13 % of the population requiring treatment globally [2, 44, 46, 55, 56].

The global IDoP elimination agenda will require strengthening community, national and regional leadership and commitment to rapidly increase funding and fostering integrated multidisciplinary and inter-sectorial policies. Furthermore, scaling-up of high-impact MDA coverage of available drugs, development and implementation of new, effective vaccines and other novel approaches and tools are needed in addressing the geographical complexity of the panoply of different diseases including malaria, river blindness, LF and schistosomiasis [56, 57]. This would mean enlarging the use of geographical information systems (GIS) and other advanced cutting-edge technologies for general surveillance and monitoring, including risk mapping of vector and parasite hotspots and studying reservoir resurgence and drug resistance. Improving local and cross-border malaria early-warning signals and surveillance systems is imperative in providing new information on potential epidemiologic transitions, crucial for quick response to any potential resurgence of vector density and competence potentially leading to disease outbreaks. These approaches will contribute to new evidence-based information needed in adapting effective health financing and programming to effective local and national programmes and interventions. Hence, consolidating and refining the laudable gains and lessons learnt from cost-effective therapeutic discoveries should contribute to the continuous pharmacovigilance associated with adverse reactions (ADR) of existing therapies leading to improved access to quality care services, procurement and supply of quality medicines and supplies needed in boosting the momentum of the elimination of IDoP.

The pharmacological and therapeutic paradigm shift discussed here calls for further, strong investments in research and development in the field of drugs and vaccines creating pipelines of new products capable of tackling the challenge of rapid emergence and spread of vectors, parasites and drug resistance. Timely, evidence-based and cost-effective operational approaches and solutions for IDoPs and NTDs are required for dealing with the rise and spread of insecticide resistance, and the environmental impact of climate change. Likewise, development of diagnostics with improved sensitivity and specificity as well as preventive therapeutics and efficient information communication/dissemination mechanisms underscore the quest for novel and innovative approaches. Leveraging on lessons learnt, efficient and integrated intersectoral partnerships as well as collaboration in the development of needed, new diagnostics, drugs and vaccines are much needed. So are also proven and innovative community-based programmes with respect to ownership in health systems, surveillance and new opportunities in elimination interventions packages and eventually in moving forward eradication of IDoP worldwide.

## Abbreviations

IDoP: Infectious Diseases of Poverty
ACT: Artemisinin Combination Therapy
ComDT: Community Directed Treatment
SDGs: Sustainable Development Goals
MDGs: Millennium Development Goals
LMICs: Low and Middle Income Countries
GMAP: Global Malaria Action Plan
GMECP: Global Malaria Eradication campaign programs
OEPA: Onchocerciasis Elimination Program of the Americas
PAHO: Pan American Health Organization
RBM: Roll Back Malaria
PRDs: Poverty-related diseases
NIDs: Neglected infectious diseases
MDA: Mass drug administration
APOC: African Programme for Onchocerciasis Control
DALYs: Disability adjusted-life years
maiERA: Malaria Eradication Research Agenda
MESA: Malaria Elimination Scientific Alliance
UNICEF: United Nations Children Emergency Funds
WHO: World Health Organization
TDR: Tropical Diseases Research
